# A Proper Increasing in the Testosterone Level May Be Associated With Better Pregnancy Outcomes for Patients With Tubal or Male Infertility During *in vitro* Fertilization/Intracytoplasmic Sperm Injection

**DOI:** 10.3389/fphys.2021.696854

**Published:** 2021-11-08

**Authors:** Zhiyan Chen, Duoduo Zhang, Zhengyi Sun, Qi Yu

**Affiliations:** National Clinical Research Center for Obstetric & Gynecologic Diseases, Department of Obstetrics and Gynecology, Peking Union Medical College Hospital, Chinese Academy of Medical Sciences & Peking Union Medical College, Beijing, China

**Keywords:** androgen, testosterone, *in vitro* fertilization (IVF), live birth rate, intracytoplasmic sperm injection (ICSI)

## Abstract

We aimed to investigate the relationship between testosterone (T) levels and pregnancy outcomes in patients with tubal or male infertility at different times during *in vitro* fertilization (IVF)/intracytoplasmic sperm injection (ICSI) cycles. Patients with tubal or male infertility and normal androgen levels undergoing IVF/ICSI were consecutively recruited. We performed a longitudinal analysis of T levels at three time points (i.e., T0: baseline, T1: trigger day, and T2: day after the trigger day) in three groups with different pregnancy outcomes (i.e., group 1: no pregnancy; group 2: clinical pregnancy but no live birth; and group 3: live birth) as repeated measurement data using linear mixed-effects models. We also plotted fitted curves depicting the relationship between T levels and a number of oocytes retrieved at different time points and identified the inflection points of the curves. In total, 3,012 patients were recruited. Groups 1 and 3 had improvements in T levels at the three time points. After refitting, the slope in group 3 was significantly higher than that in group 1 (*P* = 0.000). Curves that reflected the association between T levels and numbers of retrieved oocytes presented an upward trend before a certain inflection point, after which the curves had no obvious changes or fell with increasing T levels. The inflection points for T0, T1, and T2 were calculated as 0.45, 0.94, and 1.09, respectively. A faster upward trend in T levels might be associated with better pregnancy outcomes. Within a range lower than a T level inflection point, more oocytes and embryos could be obtained with increasing T levels.

## Introduction

Androgens, a category of sex steroid hormones, play an essential role in the endocrine and reproductive systems of women. The androgens that can be detected in the blood circulation of females include testosterone (T) (Gougeon, 1996), dihydrotestosterone, and pro-androgens such as dehydroepiandrosterone (DHEA) sulfate, DHEA, and androstenedione. These hormones activate and exert effects on sensitive tissues *via* the androgen receptor of females, and T serves as the precursor for estradiol (E2) production (Simpson et al., 2002). In recent years, the physiology of androgen in females, which has complex effects on fertility, and its utilization in assisted reproductive technology (ART), has attracted interest from gynecologists (Simpson et al., 2000). Accumulating evidence from basic discovery research, clinical trials, and meta-analysis supports the hypothesis that androgens may have a synergistic stimulatory role with the follicle-stimulating hormone (FSH) in early follicle growth, follicle health maintenance, and follicle maturation during later stages of development (Meldrum et al., 2013).

Androgen levels, including that of T and DHEA, gradually decline with age among females aged 25–40 years (Davison et al., 2005). The addition of T or DHEA in females with poor response to recombinant FSH-induced ovarian stimulation during the *in vitro* fertilization (IVF) process has been broadly undertaken by medical centers (Gleicher and Barad, 2011). In contrast, high levels of androgens can prevent follicle maturation and even harm follicle development. Hyperandrogenism, such as polycystic ovarian syndrome (PCOS) and congenital adrenal hyperplasia, is a significant cause of female infertility (Dumesic et al., 2015). Studies have shown that excess androgen has a detrimental impact on fecundity (Mannerås et al., 2007). Excessive androgens can cause hyper-recruitment of follicles in the ovaries, leading to impaired maturation and infertility (Walters et al., 2019). The effect of androgens on follicle maturation and pregnancy outcome varies with their levels; therefore, evaluation of the association between androgens and IVF outcomes has clinical value. According to previous studies, basal T levels might be related to ovarian response competence and IVF outcomes. However, T levels at different time points during IVF cycles have not yet been reported. Therefore, we designed this retrospective study and investigated the relationship between T levels and pregnancy outcomes in patients with tubal or male infertility during different time points in the IVF/intracytoplasmic sperm injection (ICSI) cycles. It is noted that different indexes of androgens, including total T, androstenedione, and free androgen index (FAI), have been proved to be feasible indicator of hyperandrogenism in clinical practice, among which FAI had the best performance (Barth et al., 2010). In this retrospective study, we chose total T levels for analysis due to technical limitation in previous years. By exploring the changes in T levels during the stimulation cycle, we aimed to identify the optimal T levels during ovarian stimulation cycles and provide clinical evidence for adding androgens in patients with poor ovarian response (POR) during the IVF process. We also expected to provide appropriate target values for androgen-lowering regimens before IVF in patients with infertility and hyperandrogenism.

## Materials and Methods

### Recruitment of Participants

The Institutional Review Board of the Peking Medical College Hospital (PUMCH) approved this retrospective observational study (No. S-K829). From July 2014 to March 2018, patients with tubal or male infertility and normal androgen levels undergoing IVF/ICSI at the PUMCH were consecutively recruited in this study. Written informed consent was obtained from all patients. Patients with hyperandrogenism, including PCOS and congenital adrenal hyperplasia, were excluded from this study. Other exclusion criteria were endometriosis; other endocrine disorders such as diabetes, pituitary dysfunction, or thyroid diseases, and a history of malignancy.

### Baseline Clinical Characteristics and Hormonal Assays

The clinical characteristics of each patient during the IVF baseline were recorded, including age, body mass index (BMI), duration of infertility, methods of ART, infertility type, gestation history, types of controlled ovarian hyperstimulation (COH) protocol [gonadotropin-releasing hormone antagonist (GnRH-a) long protocol, GnRH-a ultra-long protocol, GnRH-a short protocol, GnRH antagonist protocol, and mini-stimulation protocol], and dosage of recombinant FSH (r-FSH) and human menopausal gonadotrophin (HMG).

At three time points during the IVF/ICSI cycles, T levels were regarded as the main variables for analysis. The three time points for testing the T level are listed as follows. First, we tested the T levels on the 2nd day of menstruation before COH as the baseline values, marked as T0. Second, T levels were measured on the trigger day when the patient received human chorionic gonadotropin (hCG) for final oocyte maturation, marked as T1. The third test, marked as T2, was performed the day after hCG injection.

Baseline hormone profiles, including human FSH, E2, luteinizing hormone (LH), and prolactin (PRL), were tested on the 2nd day of the menstrual cycle. Serum levels of hormones were measured using an electrochemiluminescent immunoassay (automated Elecsys Immunoanalyzer, Beckmann, United States). The mean interassay coefficients of variation were < 5% for T, < 5% for E2, and < 8% for FSH, LH, and PRL.

### Confirmation of Primary and Secondary Outcomes

The primary outcomes of this study were clinical pregnancy rate and live birth rate. Clinical pregnancy was defined as the validation of the gestational sac and fetal heart using transvaginal ultrasound. Live birth was defined as the delivery of an infant born alive after 28 weeks of gestation. The cumulative outcomes within all the stimulation cycles of individual patients were evaluated in determining clinical pregnancy and live birth. The number of retrieved oocytes, metaphase II oocytes (MII), top-quality embryos (TQEs) on the 3rd day, and blastocyst-stage embryos were referred to as the secondary analysis outcomes. The definition of TQE was seven or more blastomeres, equally-sized blastomeres, and < 20% fragmentation on day 3 (Gardner and Schoolcraft, 1999). For patients who chose the freeze-all strategy after oocyte retrieval for various reasons, such as ovarian hyperstimulation syndrome risk and inflammation, the cryopreserved blastocysts were thawed and subsequently transplanted. The cumulative live birth rate was likewise assessed.

### Statistical Analyses

Continuous variables are described as mean ± SD, and categorical variables are expressed as percentages (%). The Student’s *t*-test was used to compare continuous variables, and Fisher’s exact test was used for categorical variables.

First, we conducted a longitudinal analysis of T levels within the cycle as repeated measurement data. The repeated measures analysis is used to examine response outcomes obtained from the same experimental unit at several time points. Longitudinal data are a typical kind of repeated measurement in which measurements are taken over time on specific individuals (Maurissen and Vidmar, 2017). Owing to the within-participant correlation of these data, linear mixed effect models were constructed using random intercept random slope models for analysis. The restricted maximum likelihood (MLE) method was used to refit the models to a straight line and calculate the regression estimates and 95% confidence intervals of the linear mixed-effects models. We performed repeated measurement analysis using a module in R (R Foundation for Statistical Computing, Vienna, Austria).^1^ The module was designed to examine the association between the risk factor (*X*) and the outcome variable (*Y*) using linear mixed effect models, where a smooth fitting curve could be specified and a random effect could be introduced. The data for the module generally had a time variable (*T*), outcome variables varying with time, while the risk factor (*X*) in turn might have an influential effect on the outcome variable (*Y*). In our analysis, we identified the T level change as *Y*, the groups with different pregnancy outcomes as *X*, and the different time points for T level examinations as the time variable.

To further identify the possible relationship of T levels at different time points with pregnancy outcomes, we plotted smooth fitting curves to fit the T levels at different time points and secondary pregnancy outcomes (i.e., the number of oocytes retrieved, metaphase II oocytes, TQE at day 3, and blastocyst-stage embryos) using the generalized additive model-based spline smoothing method, adjusting for possible-related factors (i.e., age, BMI, methods of ART, duration of infertility, infertility type, gestation history, types of COH protocol, and dosage of recombinant r-FSH and HMG) as cofounders. To further identify the inflection points of the fitted curves, we then applied segmented regression, known as piece-wise regression, to fit each interval using a distinct line segment. The log-likelihood ratio test was used to assess if a threshold exists by comparing a one-line (non-segmented) model with a segmented regression model. Statistical significance of segmented linear regression with break-point was determined using variance analysis and *F*-tests. The β coefficients of the two segments before and after the inflection point were calculated using the effect-size metric. The differences in the slopes between the two segments were evaluated using the Wald test.

For sample size estimation, we first calculated that the ratio of T level changes from T0 to T2 in the patients with no pregnancy and live birth were 0.62 and 0.50, respectively. The ratio of the two groups was 0.97. When applying the sample size of 3,012, above 95% power could be obtained with a 5% two-sided significance.

Statistical analyses were performed using R (see text footnote 1) and EmpowerStats software 2.2 (X&Y solutions, Inc., Boston, MA). Statistical significance was set at *P* < 0.05.

## Results

### Patient Characteristics and Hormone Testing

**FIGURE 1:**
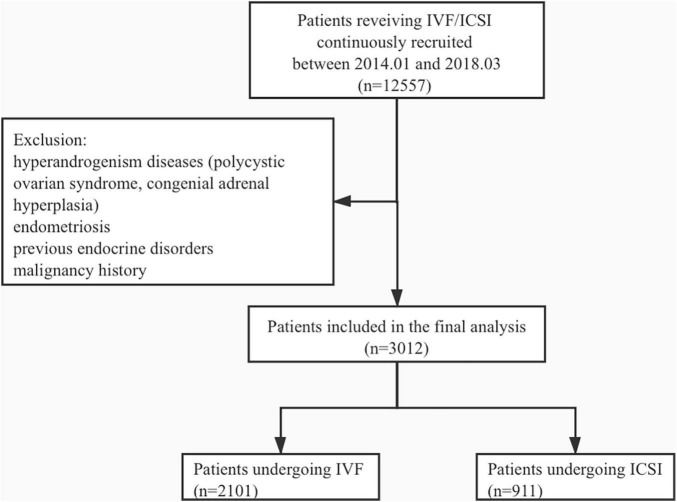
The schematic of the study flowchart.

A total of 3,012 patients undergoing IVF/ICSI were recruited for this study. The mean age of patients was 34.9 ± 4.3, and a total of 2,101 patients underwent IVF cycles, whereas 911 underwent ICSI. The study flowchart is shown in Figure 1. We divided the patients into three groups according to pregnancy outcomes as follows: group 1, no clinical pregnancy; group 2, clinical pregnancy but no live birth; and group 3, live birth. The clinical characteristics of patients from the three groups are shown in Table 1.

**TABLE 1:** Baseline characteristics of participants.

	**G1: No clinical pregnancy**	**G2: Clinical pregnancy**	**G3: Live birth**	***P*-value**
N	1,427	204	1,381	
Age (years)	35.33 ± 4.51	35.26 ± 4.10	34.43 ± 4.13	<0.001
BMI (kg/m^2^)	21.98 ± 3.18	22.40 ± 3.32	22.16 ± 3.11	0.102
Gravidity	1.15 ± 1.57	1.04 ± 1.32	0.86 ± 1.26	<0.001
Parity	0.11 ± 0.34	0.11 ± 0.32	0.07 ± 0.26	<0.001
Duration of infertility (years)	4.82 ± 3.26	5.05 ± 3.43	4.56 ± 2.79	0.025
Types of infertility				0.146
Primary infertility	804 (56.35%)	108 (53.20%)	817 (59.16%)	
Secondary infertility	623 (43.65%)	96 (46.80%)	564 (40.84%)	
Method				0.389
IVF	990 (69.45%)	151 (74.02%)	960 (69.44%)	
ICSI	437 (30.55%)	53 (25.98%)	421 (30.56%)	
Basal FSH (IU/L)	8.18 ± 4.03	7.74 ± 3.08	7.60 ± 3.33	<0.001
Basal LH (IU/L)	4.40 ± 3.55	4.12 ± 2.11	4.21 ± 2.67	0.166
Basal PRL (ng/ml)	17.92 ± 9.50	18.63 ± 10.19	17.82 ± 8.62	0.503
Basal E2 (pg/ml)	50.66 ± 31.17	49.93 ± 25.82	51.55 ± 34.40	0.675
Basal T (ng/ml)	0.48 ± 0.78	0.56 ± 1.49	0.45 ± 0.43	0.104
Total consumption of r-FSH (dose)	36.11 ± 12.60	32.72 ± 9.84	30.16 ± 16.11	<0.001
Total consumption of HMG (dose)	5.19 ± 3.71	5.08 ± 3.89	4.95 ± 4.05	0.002
COH protocol				0.009
GnRH-a long protocol	896 (62.79%)	127 (62.25%)	960 (69.51%)	
GnRH-a ultra-long protocol	30 (2.10%)	7(3.43%)	37 (2.68%)	
GnRH-a short protocol	2 (0.01%)	0 (0.00%)	2 (0.14%)	
GnRH-ant protocol	496 (34.76%)	70 (34.31%)	382 (27.66%)	
Mini-stimulation protocol	3 (0.21%)	0 (0.00%)	0 (0.00%)	

*BMI, body mass index; IVF, in vitro fertilization; ICSI, intracytoplasmic sperm injection; FSH, follicle-stimulating hormone; E2, estrogen; LH, luteinizing hormone; PRL, prolactin; T, testosterone; GnRH-a, gonadotropin-releasing hormone agonist; GnRH-ant, gonadotropin-releasing hormone antagonist; COH, controlled ovarian hyperstimulation; r-FSH, recombinant FSH; HMG, human menopausal gonadotrophin.*

### Longitudinal Analysis of the Repeated Measurement of T Levels at Three Time Points

**FIGURE 2:**
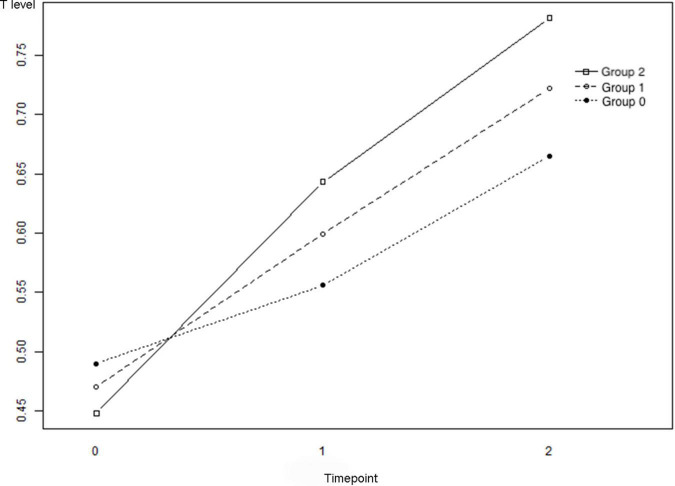
The changes of the testosterone (T) levels of Groups 1–3. Group 1: no clinical pregnancy; Group 2: clinical pregnancy and no live birth; and Group 3: live birth. T0: T levels at baseline, T1: T levels on the trigger day, and T2: T levels on the day after the trigger day.

The changes in T levels among the three groups at the baseline, trigger day of hCG administration, and the day after hCG uptake are illustrated in Figure 2. Table 2 describes the results of a comparison of T levels at different time points. For groups 1 and 3, the lines presented an overall upward trend, and improvements in T levels were observed over time [group 1: *P*(T1 - T0) = 0.000 and *P*(T2 - T0) = 0.0001; group 3: *P*(T1 - T0) = 0.000 and *P*(T2 - T0) = 0.000]. The slope of T0 - T2 in group 3 after refitting using the MLE method was significantly higher than that in group 1 (*P* = 0.000), indicating that the upward trend of T levels in live births was significantly faster than that among participants with no clinical pregnancies.

**TABLE 2:** Repeated measurement analysis of androgen levels at three time points with different IVF outcomes using linear mixed effect models.

	**T0(ng/ml)**	**T1(ng/ml)**	**T2(ng/ml)**	P^*^	**T1-T0**		**T2-T0**		**Refitting**	
					**B^a^**	**P**	**B^b^**	**P**	**B^c^**	**P**
**G1** (*n* = 1,427)	0.48 ± 0.78	0.60 ± 0.25	0.72 ± 0.38	0.104	**0.0664 ± 0.0165**	**0.000**	**0.175 ± 0.0165**	**0.000**	/	/
**G2** (*n* = 204)	0.56 ± 1.49	0.58 ± 0.24	0.71 ± 0.28	0.644	0.0626 ± 0.0467	0.179	0.0773 ± 0.0467	0.098	0.0270 ± 0.0241	0.262
**G3** (*n* = 1,381)	0.45 ± 0.43	0.60 ± 0.23	0.73 ± 0.33	0.674	**0.129 ± 0.0234**	**0.000**	**0.159 ± 0.0234**	**0.000**	**0.0542 ± 0.0121**	**0.000**

*P-value of comparing androgen levels at three time points.

*^a^Regression estimate of T1 - T0 changes, values in bold have statistical significance.*

*^b^Regression estimate of T2 - T0 changes, values in bold have statistical significance.*

*^c^Regression estimate of comparing the refitting lines of G1 with G0 and G2 with G0, values in bold have statistical significance.*

*T, testosterone.*

*T0: T levels at baseline, T1: T levels on the trigger day, and T2: T levels on the day after the trigger day.*

### Fitted Curves on the Relationship Between T Levels and Pregnancy Outcomes

**FIGURE 3:**
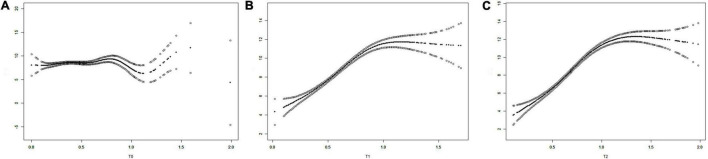
The fitting curves showing the relationship of the T levels and the numbers of oocytes retrieved [**(A)** T0, **(B)** T1, and **(C)** T2). T0: T levels at baseline, T1: T levels on the trigger day, and T2: T levels on the day after the trigger day.

We plotted the three fitted curves to illustrate the association between T levels and the number of retrieved oocytes at the three time points (T0, T1, and T2) (Figure 3). In the beginning, all curves had an upward trend, and after a certain inflection point, the curves showed no obvious changes or fell with increasing T levels. The inflection points for T0, T1, and T2 were calculated as 0.45, 0.94, and 1.09, respectively. The differences in the slopes before and after the inflection points were significant for the three curves [*P*(T0) = 0.0480, *P*(T1) < 0.0001, and *P*(T2) < 0.0001] (Table 3).

**TABLE 3:** The analysis of the inflection points and the effect sizes of the curves reflecting the association of T levels and numbers of oocytes retrieved.

	**T0**	**T1**	**T2**
Inflection point (K) (ng/ml)	0.45	0.94	1.09
β1 (95%CI) < k^*^	1.86 (0.09, 3.63)	8.92 (7.96, 9.68)	9.65 (8.96, 10.34)
P (β1)	0.0390	<0.0001	<0.0001
β2 (95%CI) > k^**^	0.03 (−0.21, 0.28)	−0.37 (−2.52, 1.78)	−1.42 (−2.20, −0.65)
P (β2)	0.7980	0.7352	0.0003
Difference for (β2-β1)	−1.83(−3.64, −0.02)	−9.19 (−11.76, −6.62)	−11.07 (−12.23, −9.91)
P (β2-β1)	0.0480	<0.0001	<0.0001

*β coefficient of the section before the inflection point.

**β coefficient of the section after the inflection point.

*T, testosterone.*

*T0: T levels at baseline, T1: T levels on the trigger day, and T2: T levels on the day after the trigger day.*

According to these results, we can conclude that at the baseline, the number of retrieved oocytes increases with T levels when the T level was < 0.45 ng/ml but was not associated with the T levels when the T level was > 0.45 ng/ml. On the trigger day, the number of retrieved oocytes increased with T levels when the T level was < 0.94 ng/ml but was not associated with the T levels when the T level was > 0.94 ng/ml. On the day after hCG administration, the value of T level is 1.07 when the numbers of retrieved oocytes start to decline with increasing T. The results of the comparison of pregnancy outcomes between patients with T levels lower than and higher than the inflection points during the three time points are shown in Table 4. Significant differences were detected in the secondary outcome variables, indicating that T levels higher than the inflection point during the three time points were associated with more acquired oocytes and embryos.

**TABLE 4:** Comparisons of pregnancy outcomes between patients with T levels ≤ and > the inflection point at the three time points.

	**T0**			**T1**			**T2**		
**Testosterone (ng/ml)**	**≤0.45**	**>0.45**	***P***	**≤0.94**	**>0.94**	***P***	**≤1.09**	**>1.09**	***P***
N	1,809	1,195		2,761	251		2,704	308	
No. of oocytes retrieved	8.22 ± 4.97	8.87 ± 5.15	<0.001	8.17 ± 4.92	11.96 ± 5.08	<0.001	8.06 ± 4.87	12.21 ± 5.04	<0.001
No. of MII oocytes	7.04 ± 4.71	7.65 ± 4.87	<0.001	7.00 ± 4.64	10.39 ± 5.10	<0.001	6.90 ± 4.58	10.61 ± 5.12	<0.001
No. of two-pronuclear zygotes	6.35 ± 4.65	6.88 ± 4.81	<0.001	6.31 ± 4.58	9.36 ± 5.26	<0.001	6.23 ± 4.52	9.50 ± 5.36	<0.001
No. of cleavage-stage embryos	6.31 ± 4.66	6.86 ± 4.82	<0.001	6.27 ± 4.60	9.36 ± 5.25	<0.001	6.19 ± 4.53	9.49 ± 5.36	<0.001
No. of TQE on the 3rd day	0.81 ± 1.27	0.84 ± 1.23	0.463	0.80 ± 1.24	1.10 ± 1.37	<0.001	0.77 ± 1.22	1.25 ± 1.50	<0.001
No. of blastocyst-stage embryos	1.66 ± 2.54	1.95 ± 2.68	<0.001	1.65 ± 2.50	3.12 ± 3.22	<0.001	1.61 ± 2.45	3.21 ± 3.31	<0.001

*T, testosterone, MII, metaphase II; TQE, top-quality embryo.*

*T0: T levels at baseline, T1: T levels on the trigger day, and T2: T levels on the day after the trigger day.*

The fitted curves and inflection point calculations of the T levels and other outcomes, including numbers of metaphase II (MII) oocytes, numbers of TQEs, numbers of blastocyst-stage embryos, TQE formation rate, and blastocyst formation rate, are shown in Supplementary Figures 1–5. The fitting curves presented a similar trend with that of T levels and oocytes retrieved with corresponding inflection points, except for that of T levels and TQE formation rate.

## Discussion

In this study, we found that for patients with tubal or male infertility who underwent IVF/ICSI, the cumulative live birth rate was higher among those who had a faster T level upward trend from the baseline to the trigger day. By examining the relationship between T level changes and the numbers of retrieved oocytes, we found that the highest level of oocyte retrieval rates can be acquired when the T levels reach 0.45 ng/ml at the baseline, 0.94 ng/ml on the trigger day, and 1.09 ng/ml on the day after hCG administration. Therefore, we hypothesized that a proper increase in T levels during ovarian hyperstimulation might increase the number of retrieved oocytes and have a positive impact on IVF outcomes.

In healthy females, androgens are a category of essential hormones that are highly involved in the promotion of follicular development by enhancing follicle recruitment and growth (Vendola et al., 1998), as well as increasing insulin-like growth factor 1 expression in the ovary (Vendola et al., 1999). Some animal studies have also shown that androgens are beneficial in follicular development through their promotion of preantral and small antral follicles in a dose-dependent manner (Shorakae et al., 2014; Lebbe et al., 2017). There is also clinical evidence indicating that androgen levels are positively correlated with ovarian response and may predict IVF outcomes (Luo et al., 2014; Sun et al., 2014). In contrast, the overexpression of androgens in patients with PCOS and other hyperandrogenic diseases can induce adverse effects on the preovulatory follicles, leading to anovulation and infertility (Dilaver et al., 2019; Owens et al., 2019). Androgens are also likely to play a role in the success rate of IVF in terms of their double-edged impact on follicle development and fertility. With the increasing use of androgen pretreatment to improve the ovarian response to hyperstimulation in patients with POR, a comprehensive assessment of the role of androgens in females undergoing IVF is needed.

The POR is a major cause of IVF failure. The addition of exogenous androgens or androgen-modulating agents in patients with POR who are undergoing IVF has been broadly utilized in clinical settings (Montoya-Botero et al., 2019). Recent studies have also focused on the pretreatment effects of androgens in patients with POR before undergoing IVF. However, contradictory results have been reported; some studies confirmed the efficacy of androgens in enhancing the live birth rate (Bosdou et al., 2016; Doan et al., 2017; Saharkhiz et al., 2018), whereas some negated this conclusion (Sipe et al., 2010; Bosdou et al., 2016). This contrast could be partly accounted for by the differences in study populations and the timing and duration of androgen pretreatment. In addition, the androgen level changes after the addition of androgens, and the association between androgen changes and IVF outcomes have not been explored.

For females undergoing IVF without endocrine abnormalities, the role of T levels in predicting IVF outcomes has not yet been established. One study on basal T levels in females with normal ovarian reserve indicated that a low T level might be relevant to the inadequate ovarian response during IVF (Qin et al., 2011). John et al. also suggested that a T level ≤ 20 ng/dl might be correlated with poor IVF outcomes, but other studies have refuted the predictive role of T (Barbieri et al., 2005; Walters et al., 2008). Furthermore, changes in androgen levels were not discussed in these studies.

To the best of our knowledge, this is the first study to explore the relationship between IVF outcomes and the T changes at different time points in the IVF cycles. In this study, we found that patients with a faster change in T levels from baseline to trigger day were more likely to achieve good IVF outcomes. This study may also explain the differential treatment efficacy of androgen pretreatment in patients with POR, as changes in T levels might influence outcomes. We planned to investigate androgen changes with pregnancy outcomes in patients receiving androgen pretreatment before IVF through further prospective cohort studies. Based on the results of this study, a reference goal of T reduction before undertaking IVF could be obtained for patients with hyperandrogenism.

This study has several limitations. It is noted that we did not use FAI for analysis, which had superior performance in determining hyperandrogenism for females than the total T according to Barth et al. (2010). Regrettably, the sex hormone-binding globulin (SHBG) examination has not been implemented regularly at our institution until 2020. As a consequence, in this study, the majority of the patients did not get SHBG and FAI data, which constitute the main limitation of our investigation. Considering that total T was proved to have a relatively acceptable accuracy in representing the androgen level of females (Barth et al., 2010), in this study, we think it can be a feasible alternative for FAI. In addition, FAI has been introduced as an essential indicator in our subsequent prospective and perspective studies. Moreover, in this study, only early follicular phase FSH was used to evaluate the ovarian functional reserve of the patient. Anti-Müllerian hormone (AMH) and antral follicle count (AFC) were not included, which were also not tested for the patients in our institution during the recruiting time. Other limitations include the retrospective and single-center design of the study. As a retrospective study, selection and recall biases were inevitable. We attempted to minimize recall bias by adjusting confounding variables and extracted the data from a computerized database. Also, this single-center study had a limited number of patients and IVF cycles. In future studies, the sample size should be enlarged to further validate our conclusion. Finally, we excluded patients with hyperandrogenism and focused mainly on patients with tubal or male infertility. Therefore, studies on androgen changes in patients with endocrine disorders are necessary.

## Conclusion

By exploring the changes in T levels during various time points of the IVF/ICSI cycles, we found that the faster upward trend of the T levels might be associated with better pregnancy outcomes. Moreover, pregnancy outcomes are positively associated with T levels, within a certain range. Therefore, a proper increase in T levels might be beneficial for enhancing ovarian responses and IVF outcomes.

## Data Availability Statement

The original contributions presented in the study are included in the article/Supplementary Material, further inquiries can be directed to the corresponding author/s.

## Ethics Statement

The Institutional Review Board approved the retrospective observational study of Peking Medical College Hospital. The patients/participants provided their written informed consent to participate in this study.

## Author Contributions

DZ collected and validated the patient data. ZC analyzed and interpreted the patient data and was a major contributor in writing the manuscript. ZS and QY supervised this study and revised the manuscript. All authors read and approved the final manuscript.

## Conflict of Interest

The authors declare that the research was conducted in the absence of any commercial or financial relationships that could be construed as a potential conflict of interest.

## Publisher’s Note

All claims expressed in this article are solely those of the authors and do not necessarily represent those of their affiliated organizations, or those of the publisher, the editors and the reviewers. Any product that may be evaluated in this article, or claim that may be made by its manufacturer, is not guaranteed or endorsed by the publisher.

## Footnotes

1
http://www.R-project.org
