# Supplementary material for: A Proper Increasing in the Testosterone Level May Be Associated With Better Pregnancy Outcomes for Patients With Tubal or Male Infertility During in vitro Fertilization/Intracytoplasmic Sperm Injection
Source: Front Physiol. 2021 Nov 8;12:696854. doi: 10.3389/fphys.2021.696854 (PMC8606517; doi:10.3389/fphys.2021.696854)
Supplement: Supplementary file 1 [file Table_1.DOCX]

### Supplementary materials

### Fitting curves of the relationship between androgen levels and other ovarian stimulation outcomes

We plotted three fitting curves to depict the association of androgen levels and other ovarian stimulation outcomes apart from numbers of retrieved oocytes at the three-time points (T0, T1, T2), including numbers of metaphase II(MII) oocytes, numbers of top quality embryos (TQEs), numbers of blastocyst-stage embryos, TQE formation rate and blastocyst formation rate. (Figure S1-5). The fitting curves presented similar trend with that of T levels and oocytes retrieved with corresponding inflection points, except for that of T levels and TQE formation rate (Table S1).

**Table S1.** The analysis of the inflection points and the effect sizes of the curves reflecting the association of T levels and the secondary pregnancy outcomes

| T levels and numbers of MII oocytes |  |  |  |
| --- | --- | --- | --- |
|  | **T0** | **T1** | **T2** |
| Inflection point (K) (ng/mL) | 0.46 | 0.89 | 1.09 |
| β1 (95%CI) <k^*^ | 0.87 (-0.77, 2.50) | 7.20 (6.22,8.13) | 7.76 (7.03, 8.49) |
| P (β1) | 0.2977 | <0.0001 | <0.0001 |
| β2 (95%CI) >k^**^ | 0.04 (-0.15, 0.24) | 0.07 (-1.96, 2.11) | -1.26 (-1.84, -0.68) |
| P (β2) | 0.6672 | 0.9446 | <0.0001 |
| Difference for (β2-β1) | -0.82 (-2.49, 0.84) | -7.13 (-9.63, -4.63) | -9.02 (-10.05, -8.00) |
| P (β2-β1) | 0.3316 | <0.0001 | <0.0001 |

| T levels and numbers of TQE | |  | |  | |  | | |
| --- | --- | --- | --- | --- | --- | --- | --- | --- |
|  | | **T0** | | **T1** | | **T2** | | |
| Inflection point (K) (ng/mL) | | 0.28 | | 0.87 | | 1.03 | | |
| β1 (95%CI) <k^*^ | | 0.48 (-0.57, 1.53) | | 1.09 (0.84, 1.34) | | 1.12 (0.91, 1.34) | | |
| P (β1) | | 0.3713 | | <0.0001 | | <0.0001 | | |
| β2 (95%CI) >k^**^ | | 0.01 (-0.06, 0.07) | | -0.25 (-0.78, 0.29) | | -0.16 (-0.34, -0.02) | | |
| P (β2) | | 0.8266 | | 0.3666 | | 0.078 | | |
| Difference for (β2-β1) | | -0.47 (-1.53, 0.59) | | -1.33 (-2.00, -0.67) | | -1.29 (-1.59, -0.98) | | |
| P (β2-β1) | | 0.3830 | | <0.0001 | | <0.0001 | | |
| T levels and numbers of blastocyst-stage embryos | |  | |  | |  | |  |
|  | | **T0** | | **T1** | | **T2** | |  |
| Inflection point (K) (ng/mL) | | 0.74 | | 0.94 | | 1.16 | |  |
| β1 (95%CI) <k^*^ | | 0.45 (-0.13, 1.03) | | 2.72 (2.23, 3.21) | | 2.74 (2.34, 3.14) | |  |
| P (β1) | | 0.1269 | | <0.0001 | | <0.0001 | |  |
| β2 (95%CI) >k^**^ | | 0.03 (-0.11, 0.17) | | 0.40 (-1.01, 1.82) | | -0.54 (-0.90, -0.18) | |  |
| P (β2) | | 0.7101 | | 0.5758 | | 0.0031 | |  |
| Difference for (β2-β1) | | -0.42 (-1.04, 0.19) | | -2.31 (-3.95, -0.68) | | -3.28 (-3.87, -2.69) | |  |
| P (β2-β1) | | 0.1790 | | 0.0054 | | <0.0001 | |  |
| T levels and TQE formation rate | |  | |  | |  | |  |
|  | | **T0** | | **T1** | | **T2** | |  |
| Inflection point (K) (ng/mL) | | 0.22 | | 0.32 | | 0.36 | |  |
| β1 (95%CI) <k^*^ | | 0.22 (-0.02, 0.47) | | 0.30 (0.08, 0.52) | | 0.38 (0.17, 0.60) | |  |
| P (β1) | | 0.0780 | | 0.0067 | | 0.0005 | |  |
| β2 (95%CI) >k^**^ | | 0.00 (-0.01, 0.02) | | 0.01 (-0.02, 0.05) | | 0.01 (-0.01, 0.02) | |  |
| P (β2) | | 0.4594 | | 0.3573 | | 0.4872 | |  |
| Difference for (β2-β1) | | -0.22 (-0.47, 0.03) | | -0.29 (-0.52, -0.06) | | -0.38 (-0.59, -0.16) | |  |
| P (β2-β1) | | 0.0865 | | 0.0140 | | 0.0007 | |  |
| T levels and blastocyst formation rate | |  | |  | |  | |  |
|  | | **T0** | | **T1** | | **T2** | |  |
| Inflection point (K) (ng/mL) | | 0.78 | | 0.86 | | 1.26 | |  |
| β1 (95%CI) <k^*^ | | 0.04 (-0.01, 0.09) | | 0.18 (0.13, 0.23) | | 0.17 (0.14, 0.20) | |  |
| P (β1) | | 0.1120 | | <0.0001 | | <0.0001 | |  |
| β2 (95%CI) >k^**^ | | 0.00 (-0.01, 0.01) | | 0.05 (-0.04, 0.14) | | -0.04 (-0.08, 0.00) | |  |
| P (β2) | | 0.5930 | | 0.3010 | | 0.0796 | |  |
| Difference for (β2-β1) | | -0.04 (-0.09, 0.02) | | -0.13 (-0.25, -0.01) | | -0.21 (-0.27, -0.15) | |  |
| P (β2-β1) | | 0.1692 | | 0.0323 | | <0.0001 | |  |

*: β coefficient of the section before the inflection point

**: β coefficient of the section after the inflection point

T: testosterone

MII: Metaphase II

TQE: top quality embryo

**Figure S1**


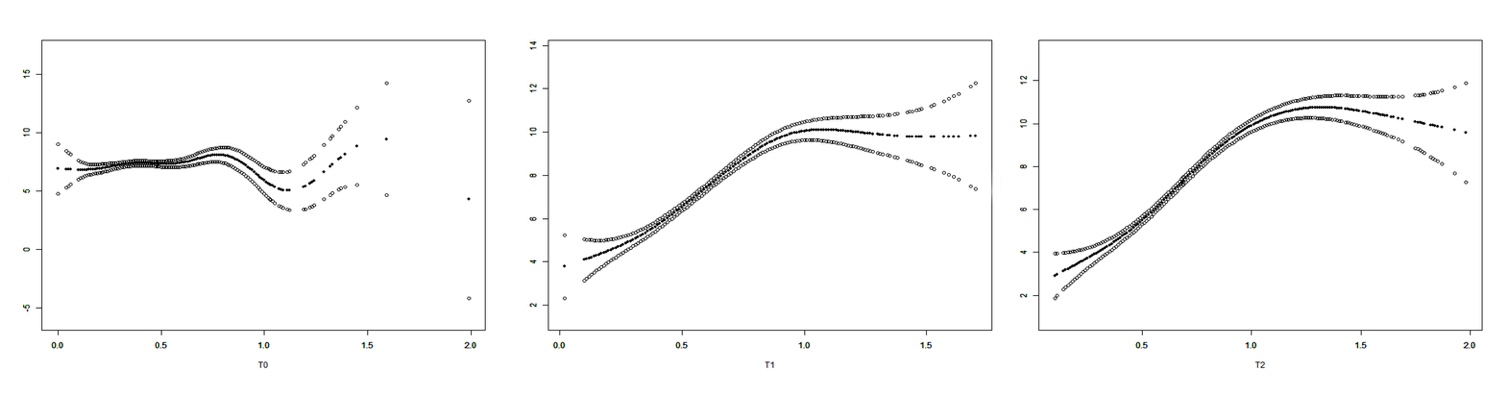


**Figure S1.** The fitting curves showing the relationship of the testosterone levels and the numbers of metaphase II (MII) oocyets. (a. T0, B. T1, C. T2)

T0: T levels at baseline, T1: T levels on the trigger day, T2: T levels on the day after the trigger day

**Figure S2**


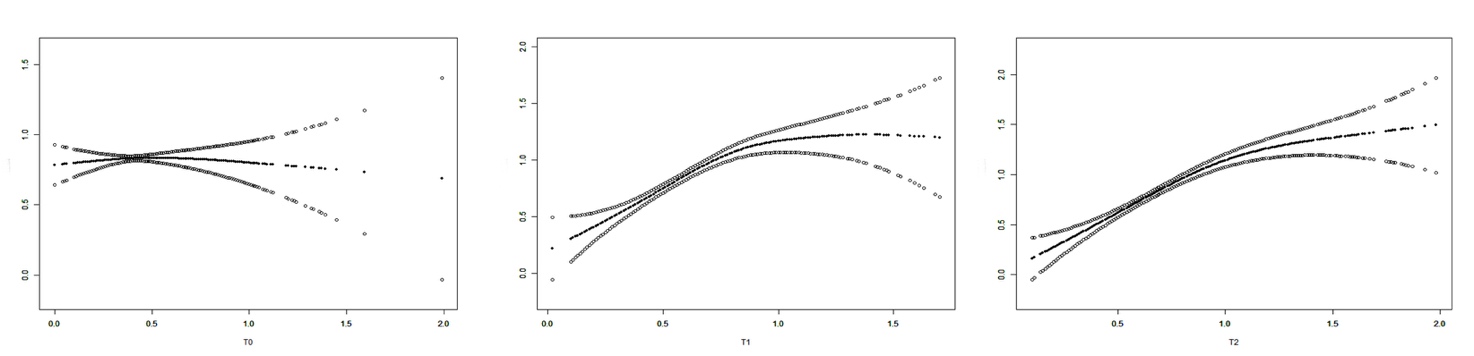


**Figure S2.** The fitting curves showing the relationship of the testosterone levels and the numbers of top-quality embryos at Day 3 (TQE[D3]). (a. T0, B. T1, C. T2)

T0: T levels at baseline, T1: T levels on the trigger day, T2: T levels on the day after the trigger day

**Figure S3**


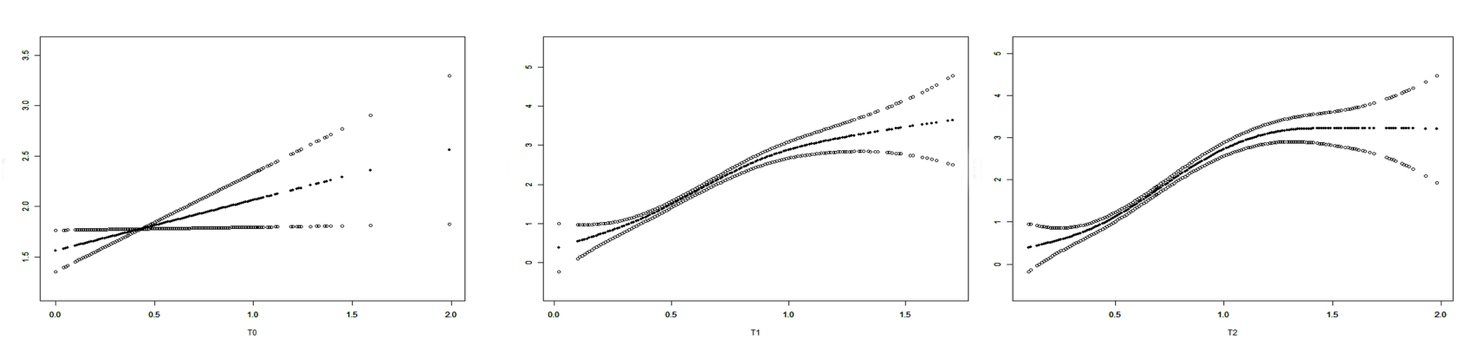


**Figure S3.** The fitting curves showing the relationship of the testosterone levels and the numbers of blastocyst-stage embryos. (a. T0, B. T1, C. T2)

T0: T levels at baseline, T1: T levels on the trigger day, T2: T levels on the day after the trigger day

**Figure S4**

**
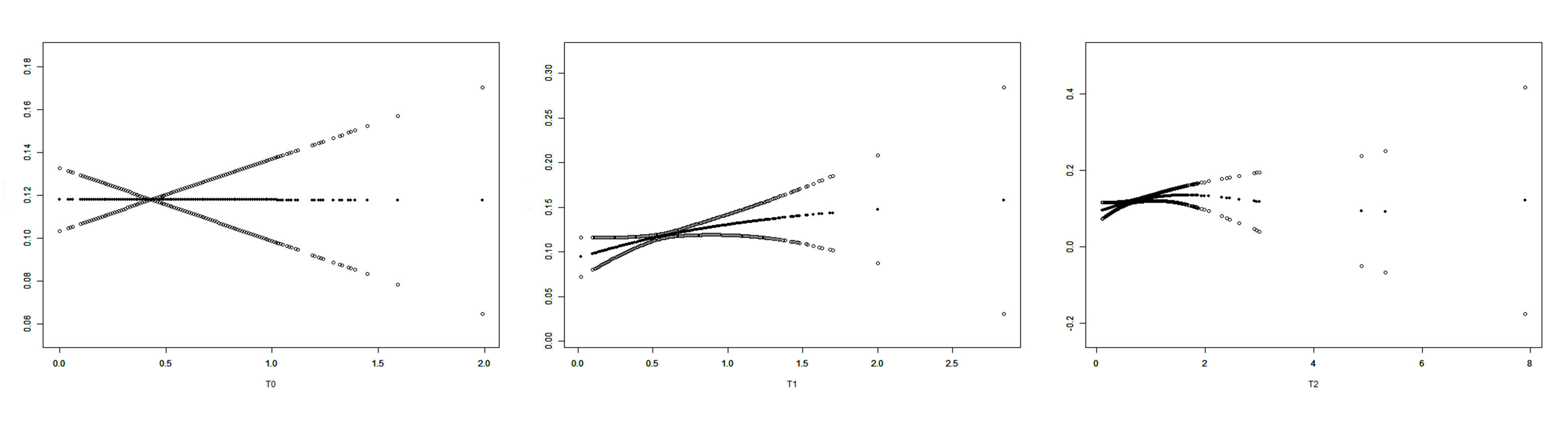
**

**Figure S4.** The fitting curves showing the relationship of the testosterone levels and the numbers of top-quality embryos (TQE) formation rate. (a. T0, B. T1, C. T2)

T0: T levels at baseline, T1: T levels on the trigger day, T2: T levels on the day after the trigger day

**Figure**
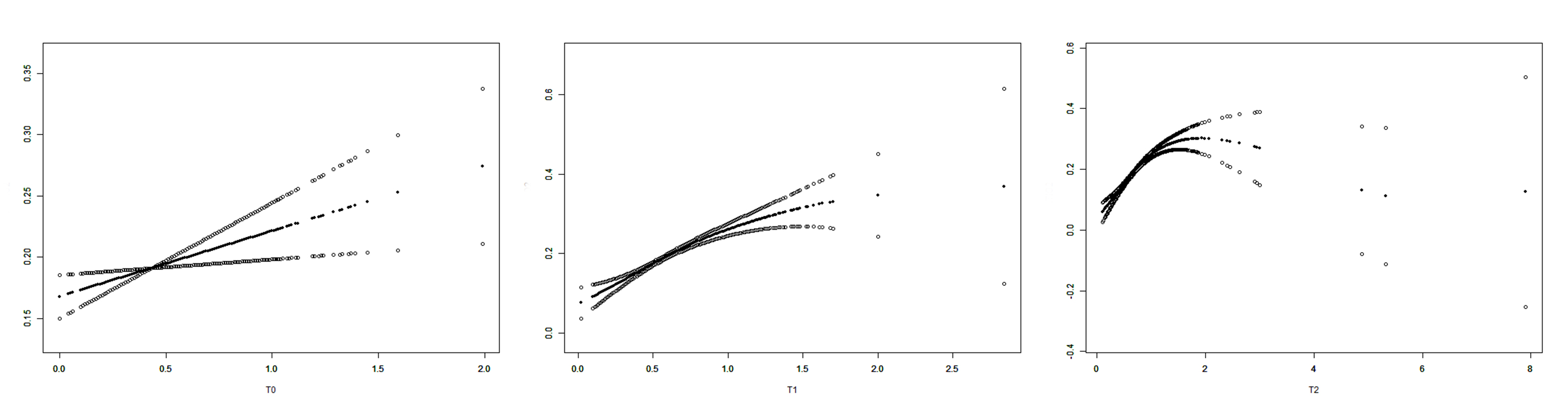


**Figure S5.** The fitting curves showing the relationship of the testosterone levels and the numbers of blastocyst formation rate. (a. T0, B. T1, C. T2)

T0: T levels at baseline, T1: T levels on the trigger day, T2: T levels on the day after the trigger day
