# Supplementary figures and images for: A Proper Increasing in the Testosterone Level May Be Associated With Better Pregnancy Outcomes for Patients With Tubal or Male Infertility During in vitro Fertilization/Intracytoplasmic Sperm Injection
Source: Front Physiol. 2021 Nov 8;12:696854. doi: 10.3389/fphys.2021.696854 (PMC8606517; doi:10.3389/fphys.2021.696854)

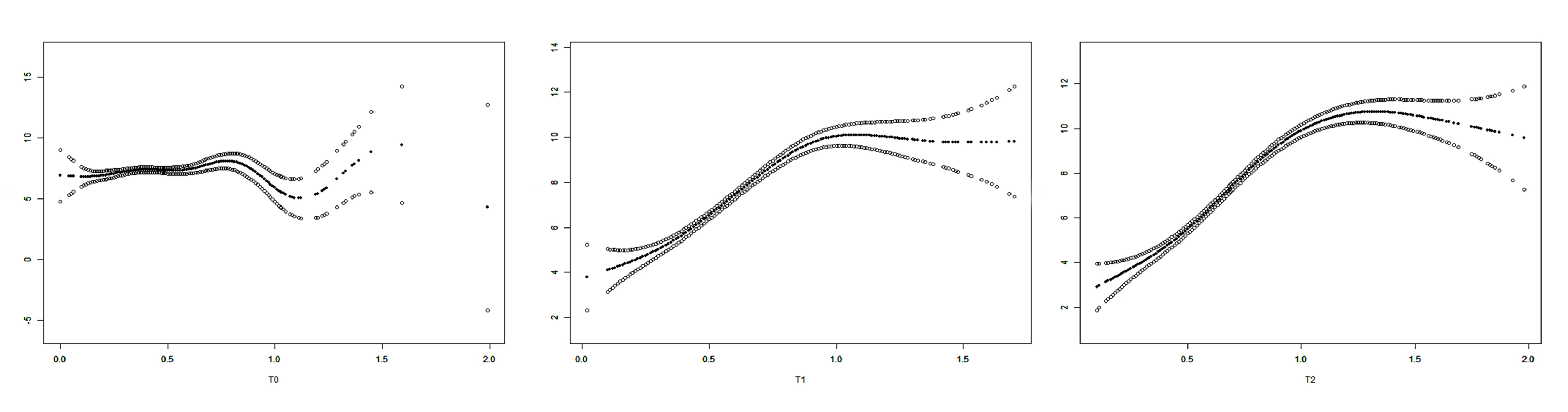

Supplement: Supplementary file 2 [file Image_1.JPEG]

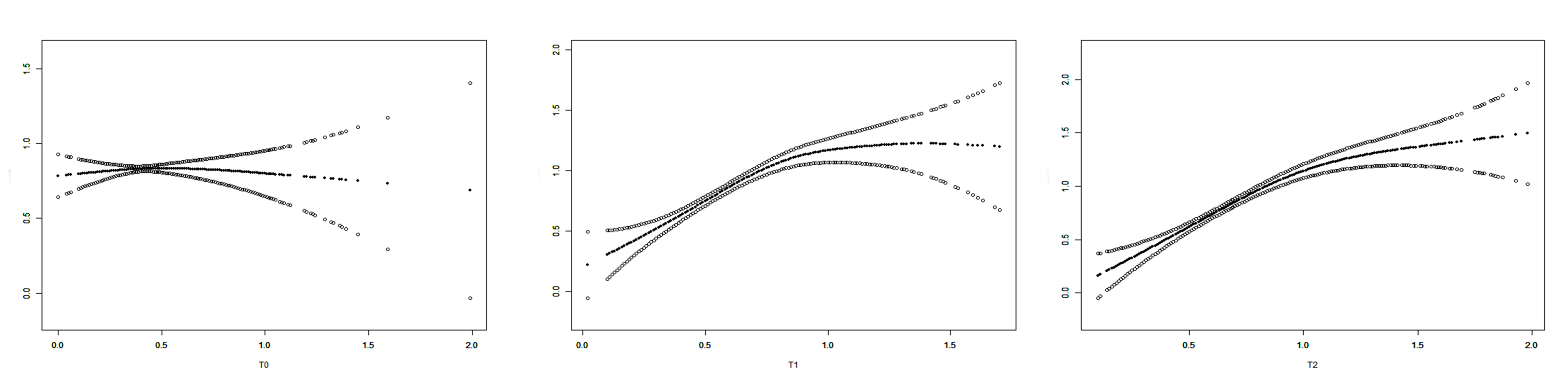

Supplement: Supplementary file 3 [file Image_2.JPEG]

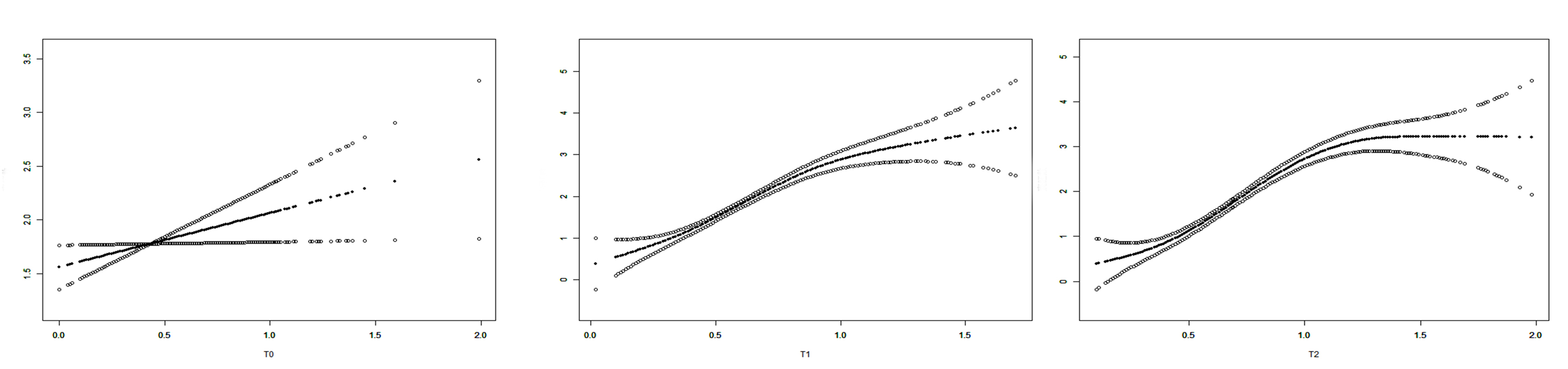

Supplement: Supplementary file 4 [file Image_3.JPEG]

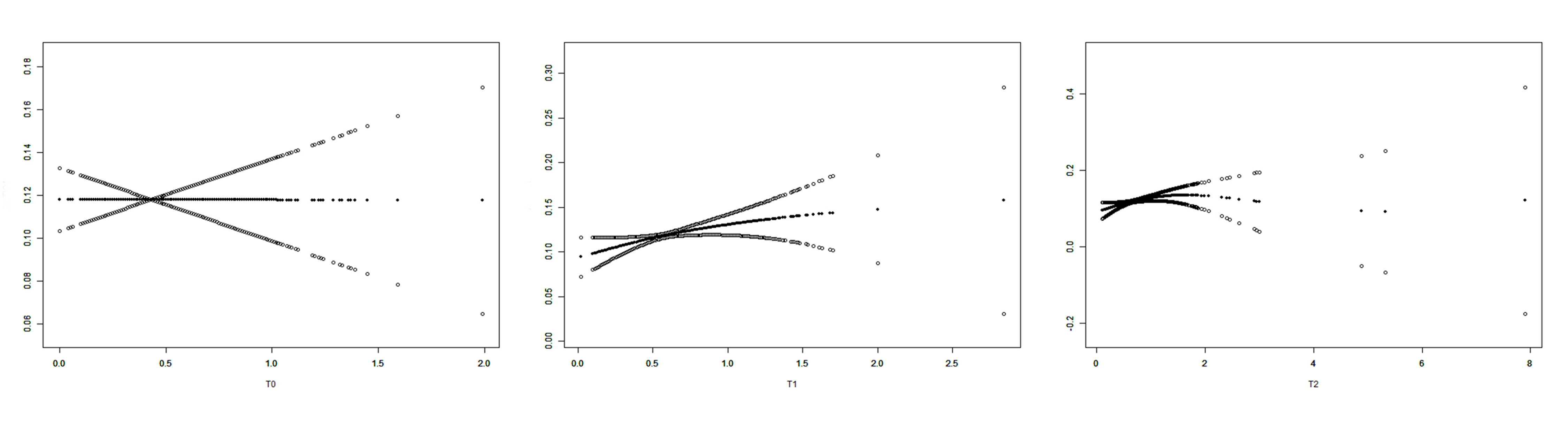

Supplement: Supplementary file 5 [file Image_4.JPEG]

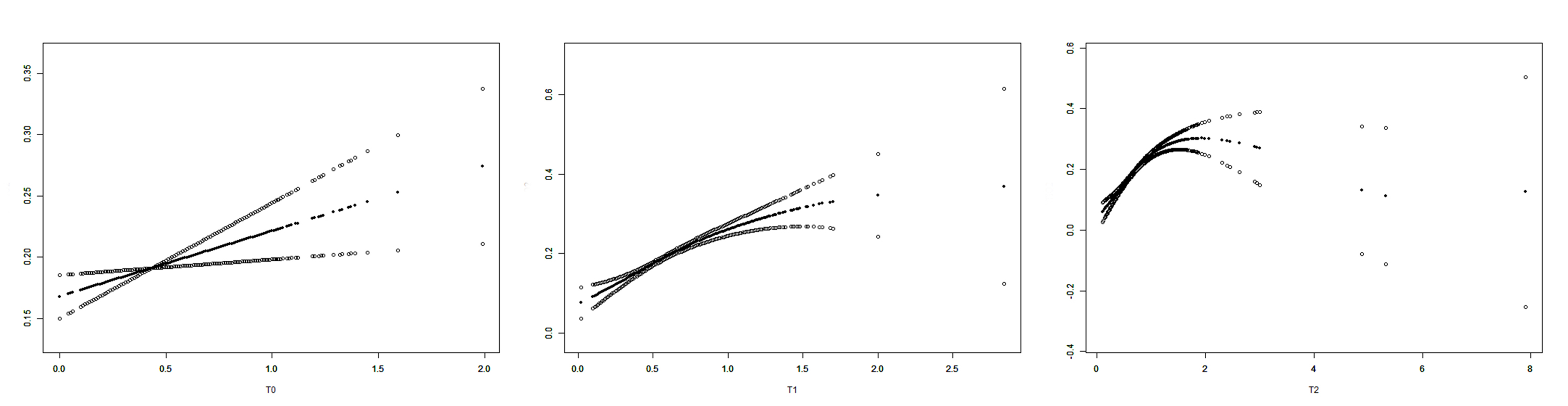

Supplement: Supplementary file 6 [file Image_5.JPEG]
